# Psychological function in the context of protracted stress during war: a multi-sample, multivariate longitudinal study

**DOI:** 10.3389/fpsyt.2025.1729795

**Published:** 2026-04-23

**Authors:** Yaakov Greenwald, Dana Katsoty, Dema Abu-Raya, Sharon Cayzer-Haller, Noa Levy, Tamar Machlev-Blank, Nitzan Shoham, Maya Benish-Weisman, Ella Daniel, Shaul Oreg, Noga Sverdlik, Ariel Knafo-Noam

**Affiliations:** 1 Department of Psychology, The Hebrew University of Jerusalem, Jerusalem, Israel; 2 The Paul Baerwald School of Social Work and Social Welfare, The Hebrew University of Jerusalem, Jerusalem, Israel; 3 Department of School Counseling and Special Education, Tel Aviv University, Tel Aviv, Israel; 4 School of Business Administration, The Hebrew University of Jerusalem, Jerusalem, Israel; 5 School of Education, Ben Gurion University of The Negev, Beersheba, Israel

**Keywords:** psychological function, stress, trauma, risk, resilience, war

## Abstract

Continuous traumatic stress has wide-ranging implications for important life outcomes across multiple domains. We present the design protocol from the first waves of one of the most comprehensive studies of the impact of one pervasive continuous traumatic stress context—war—on individuals. In this ongoing project we have been collecting quantitative and qualitative data on psychological function, risk, and resilience at different levels of influence and at various junctures during the ongoing 2023–2025 Hamas-Israel war from three samples (total N = 16,330). We present this large-scale, multi-sample, multivariate, mixed-method, longitudinal study, and showcase select, preliminary findings at different levels of analysis and in different samples (e.g., concerning war exposure, trust in institutions, and well-being). We document the design, scope, and future trajectory of the project, encouraging interdisciplinary, cross-border collaborations among researchers across diverse fields. This is important not only for understanding responses to the current conflict, but also for understanding risk and resilience in other conflict-affected regions and among populations facing continuous traumatic stress more broadly.

## Introduction

Continuous traumatic stress (CTS) describes the experience of living in contexts that present realistic, ongoing threats and dangers, such as those encountered in political conflict, war, and terrorism (1). Unlike acute traumatic exposure, CTS involves ongoing stressors that demand sustained cognitive and emotional resources. It typically involves the experience of multiple, wide-ranging adversities, that extend beyond the individual level to affect entire communities and nations (2). Its effects are thus substantial, far beyond those of acute trauma (3, 4), impacting many aspects of life, ranging from one’s personal health and behavior to economic stability and well-being of entire communities. In the present study we consider such broad implications of CTS in the context of the Hamas-Israel war that followed the October 7, 2023, terror attack on Israel. Casting a wide theoretical net, we planned and are conducting an extensive study, capturing a broad range of CTS-related events and outcomes. In doing so, we aim to lay a foundation for further research and novel insights into this critical phenomenon.

**Figure 1:**
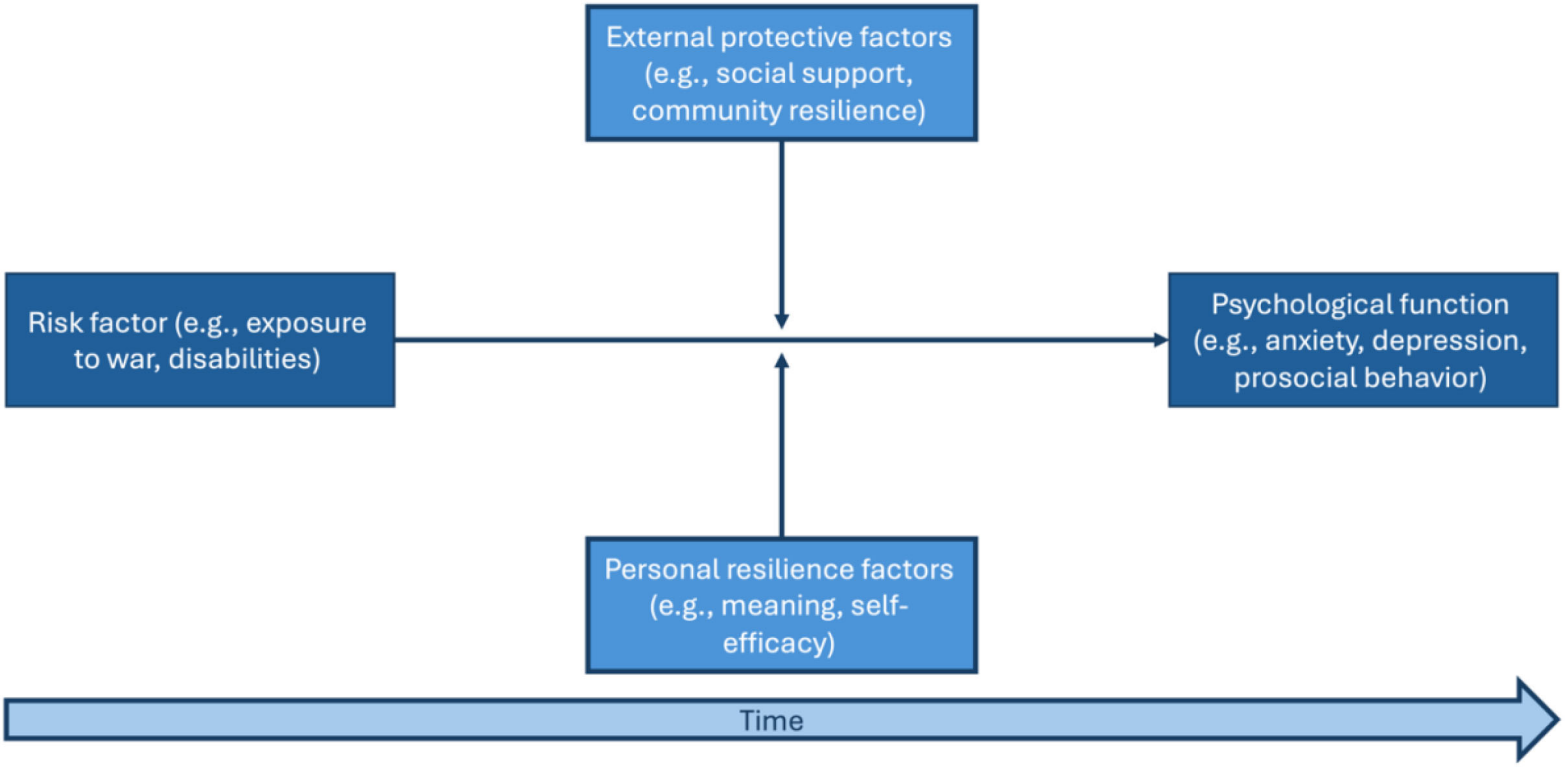
Psychological model of risk and resilience.

We were guided by a psychological model of risk and resilience whereby psychological function is the outcome of exposure to adverse circumstances, accompanied by both situational and psychological protective factors, over time (**Figure 1**). Given the scope of the phenomenon, we were further informed by Bronfenbrenner’s systems-based, bioecological model of development (5), which highlights the interplay of micro- and macro-level contexts and their effects on psychological adjustment. These contexts include intrapersonal characteristics, interpersonal factors, as well as family, community, and cultural attributes. Each component poses a distinct tier of influence, and they might mutually affect each other across time. Indeed, previous studies identified a wide range of risk and resilience factors of psychopathology in the context of CTS. Variables at the intrapersonal (attachment style, self-criticism, perceived self-responsibility, substance use), interpersonal (social support, sense of community belonging), demographic (gender, age), and socioeconomic (rural vs. urban living, SES) levels were associated with psychopathology risk (6). On this basis, we aimed to assess variables related to the individual and to different contextual levels. To address the chronosystem, the role of time in change (5), we monitored the effects of CTS and protective factors across different times during the war, as well as in longitudinal designs.

In addressing specific questions of coping, the general model of risk and resilience combined with the broad framework offered by Bronfenbrenner’s bioecological model of development may be complemented by other theoretical perspectives to allow for an in-depth examination of responses to continuous stress. To name select examples, Conservation of Resources Theory (7) emphasizes actual resource loss and gain (i.e., objective reality, rather than appraisals) under stress and trauma to understand risk and resilience in such contexts. Social Ecology of Resilience Theory (8, 9) focuses on how individuals interact with their environment to access resources that foster resilience during adversity. The Socio-interpersonal Perspective (10) integrates both interpersonal processes and contextual factors into a single model, emphasizing interpersonal processes at multiple levels (e.g., within individual, relationship, society). Theoretical perspectives that consider factors beyond risk, resilience, and posttraumatic reactions may be equally useful. Terror Management Theory (11) considers how mortality awareness in such contexts may motivate a wide range of belief and behavior. Attachment Theory (12) emphasizes how individual differences in *attachment orientation*—patterns of relational expectations and responding (13)—lead to different reactions to stress. And Schwartz’s Theory of Basic Human Values (14) identifies a universal set of basic human motivations that guide attitudes and behaviors. The combination of different theories may thus result in an interdisciplinary and holistic approach to psychological adjustment in highly adverse circumstances—such as war.

### The war context

The October 7, 2023, terrorist attack by Hamas against Israel triggered the ongoing 2023–2025 Hamas-Israel war, resulting in mass casualties and suffering across the region. As researchers based in Israel, our focus in the present study is on the Israeli population, and our description of the war-related conditions is thus centered on the Israel context. This is not intended to downplay the devastating impact of the war on Palestinians and other populations in the region, and we call for studies to investigate the psychological impact of the war in these populations. During the initial October 7 attack, Hamas infiltrated Israel under the cover of rocket bombardment and killed over 1,100 Israelis. Thousands more were wounded, many were subjected to torture and sexual violence, and 251 Israelis—including men, women, the elderly, and children—were abducted into the Gaza Strip (15). Some of the hostages were killed, one deceased hostage remains in Gaza at the time of this writing, and all others were either rescued via military operations or released during ceasefires following negotiations between Hamas and Israel. In addition to rocket fire from Gaza, Israel has faced intensive projectile and drone attacks from Lebanon, Yemen, and Iran. In the first months of the war, approximately 143,000 Israelis from the northern and southern regions were internally displaced by government instruction due to the fighting (this number does not include those who fled voluntarily) (16). Many homes were destroyed by bombardment leaving residents with nowhere to return to, and people fell upon financial troubles due to various war-related effects. In the small Israeli society, large populations were indirectly exposed to the violence through its effects on members of family and friends. Videos and testimonies depicting extreme physical and sexual violence from the October 7 attack circulated widely on social and mainstream media. Thus, even individuals not under direct threat (e.g., of invasion or rocket attacks), were indirectly exposed to death and destruction of neighboring individuals, communities, and cities (17, 18).

### The present study

To study the effects of this continuous trauma, we collected longitudinal data from three samples: (a) a panel sample, (b) a community sample, and (c) and a student sample. In each sample and at each wave, we assessed a variety of variables at different ecological levels. These include psychological (e.g., affect and emotion regulation, personality, self-efficacy, interpersonal trust, psychological distress), behavioral (e.g., pro-social behavior, news exposure, social media use), social (e.g., family relationships, social support), and demographic variables (e.g., gender, income, education, religiosity). We also examined the extent to which individuals were exposed to various war-related events (e.g., terror attacks, rocket attacks). Although a major primary outcome in contexts of CTS is trauma and mental health functioning, the primary outcome will depend on the investigation. For example, religious/spiritual change was the primary outcome in an investigation of the impact of war on religion and spirituality (19). However, it is also possible that religion may have beneficial effects on well-being in the context of CTS. Studying how individuals form opinions about war means primary outcome variables such as attitudes toward military action or hostage negotiations (20). Therefore, predictors, outcomes, and hypotheses will vary from one investigation to another. In the following sections, we present a detailed account of our study’s design and describe some preliminary results with sample analyses that address varied questions. This serves to call for cross-border, interdisciplinary collaborations among researchers of varying expertise.

## Method and analyses

### Participants and procedure

Shortly after the start of the ongoing 2023–2025 Hamas-Israel war, we launched longitudinal data collection in three main samples: (a) a panel sample, (b) a community sample, and (c) and a student sample. The vast majority of participants (respectively, 98.6%, 96.8%, and 96%) reported holding Israeli citizenship and considered themselves as Jews, which means they are from the Hebrew-speaking Israeli Jewish majority. Arab samples were also recruited with similar community and student designs (21) but they were substantially smaller, and are therefore not part of the current report. Here, we outline data collection procedures for each study sample in turn, followed by a description of our measurement tools (additional details are in the **Supplementary Material**). This research project was approved by the IRB of The Hebrew University of Jerusalem (approval numbers: IRB_2023_005, IRB_2023_054, IRB_2023_063). The research is ongoing, and the current protocol covers the first year of the war, i.e., the period from October 2023 to October 2024.

#### Panel sample

The Panel Sample comprises a representative sample of predominantly Jewish, Hebrew-speaking Israeli adults. Participants were contacted via the Panel4All research company, beginning on October 16, 2023, nine days after the war began. We requested the company to attempt to recruit a representative sample based on gender, age, and religiosity (secular, traditional, religious, and ultra-Orthodox). Indeed, the sample is quite comparable to the general population on these parameters (**Supplementary Table S1**). The relatively lower rate of participation in the oldest age bracket is likely because in this group (i.e., the older segment of the elderly) there is a relatively high rate of individuals who are unable to participate, either due to the need to participate online or due to their health status.

Because we did not know how events would unfold, and to maximize data collection at various junctures during the war, we divided the sample into subgroups, as follows. Two thirds of the respondents (66.6%) participated in Time 1 (T1) beginning on October 16, 2023, and the remaining individuals participated beginning on October 24, 2023. We randomly split the October 16 participants into two subgroups, and all three groups participated in either one or two additional waves at varying intervals in November and early December of 2023. Henceforth, the October 16 subgroups are referred to as Panel A and Panel B, and the October 24 subgroup is referred to as Panel C.

Through December, participants in each subgroup were contacted for subsequent waves if they correctly answered each of two attention checks—with one exception. We intended for Panel A (rather than Panel B) to participate in Time 2 (T2) beginning on November 12, however, due to a technical error, the survey was sent to Panel B (this group had already participated in T2). Therefore, Panel B’s Time 3 (T3) follow-up inclusion criterion for recruitment was derived from T1 rather than T2, and the interval between T2 and T3 in this group was relatively brief.

Participants from all groups were contacted for a six-month follow-up on April 16, 2024, and for a one-year follow-up on October 6, 2024. The follow-up recruitment inclusion criterion for these time points was derived from T1. Following the aforementioned criterion, eligibility rates that determined recruitment for subsequent waves ranged from 89.6% to 95.7%. Response rates (i.e., proportion of eligible participants who responded) during the first two months of the war ranged from 83.3% to 88.9%, and response rates at the six-month and one-year follow-ups ranged from 66.7% to 71.5%. Data collection for each wave during the first two months of the war spanned two to 10 days, whereas data collection for the six-month and one-year follow-ups lasted as long as 20 days. The extended data collection periods of the latter waves were to minimize attrition.

#### Community sample

In parallel to the Panel Sample, we began data collection in a convenience sample of predominantly Jewish Israeli adults—the Community Sample. Participants were contacted via links shared online beginning on October 15, 2023, eight days after the war began. To aid in data collection, we ran three paid promotions on Facebook and Instagram in 2023 (November 2–9 and 14-21, as well as November 30-December 7) and one in 2024 (April 18-27). Also, due to interest in our study, a mainstream news outlet (Mako) published a running link to the survey from February 11 through April 11. Although the survey was live for the entire duration from October 15 through May 1, participation rates varied over time based on these recruitment strategies. In addition, different recruitment methods resulted in varied demographics. Nevertheless, one could easily draw representative samples from the data set given the large overall sample size (*n* = 13,842).

Participants who were interested in being entered into a raffle in return for their participation, receiving updates about the study, or participating in future waves, were asked to provide an email address or phone number. Those who participated prior to April 6, 2024, passed each of two attention checks, and provided a valid email address (*n* = 3,543) or WhatsApp number (*n* = 250) were contacted to participate in a second wave (T2) on April 16, 2024, six months after data collection launched. A reminder to participate was sent out on April 19, and ultimately a total of 1,081 participants took part in the second wave. All who participated in T1 (i.e., by May 1), passed each of two attention checks, and provided a valid email address (*n* = 4,148) or WhatsApp number (*n* = 295) were contacted to participate in a third wave (T3) on October 7, 2024, one year after the war broke out. Email reminders to participate were distributed on October 14, October 27, and November 3, and ultimately a total of 615 participants took part in the third wave. The low response rates (28.5% at T2 and 13.8% at T3) are likely because many participants who provided contact information were interested in participating in the raffle, but not in future waves.

#### Student sample

Three and a half months after the war began, and three weeks after the universities in Israel commenced the academic year (delayed for over two months due to the war), we ran a study among students. All students at The Hebrew University of Jerusalem were contacted via the university email distribution system on January 22, 2024. Reminders to participate were sent out via the same system again on February 7 and February 13.

Following the same procedure as in the Community Study, participants who passed each of two attention checks and provided a valid email address (*n* = 963) or WhatsApp number (*n* = 46) were contacted to participate in a second wave (T2) on April 16, 2024, and a reminder to participate was sent out on April 19. Here too, the low response rate (*n* = 296; 29.3%) is likely because many participants who provided contact information were interested in participating in the raffle, but not in future waves.

#### All samples

##### Participant inclusion and data retention

Participation in the study (i.e., inclusion in the final samples) was considered if the participant answered at least one item on the Patient Health Questionnaire-4 (PHQ-4) (22). We chose this as the criterion because (a) this is a core measure—all participants across all samples completed it—and (b) it was always presented early in the survey and was therefore not overly exclusive. Yet, this did exclude participants with very few data points (i.e., those who only clicked on the link or answered a few items before dropping out). Next, we retained only one response of participants who responded more than once to the same wave. Because panel sample participants are only paid by the panel company upon survey completion, and because surveys are available to them until completion, participants may have responded more than once if their initial attempt was incomplete or technical issues (e.g., failed internet connection) prevented them from being rerouted back to the panel website. This same issue led to a few participants responding to more than one panel (e.g., Panel A and Panel C). In all cases, we retained the first response when responses were equally complete provided one response was not clearly qualitatively superior (e.g., passed versus failed attention check), otherwise we retained the more complete response. In the Community Sample and Student Sample, participants could theoretically participate multiple times (e.g., encountering the survey link on different platforms). When we were able to identify duplicate responses of community and student participants via their contact details, we retained only the first response.

##### Study timeline

**Figure 2:**
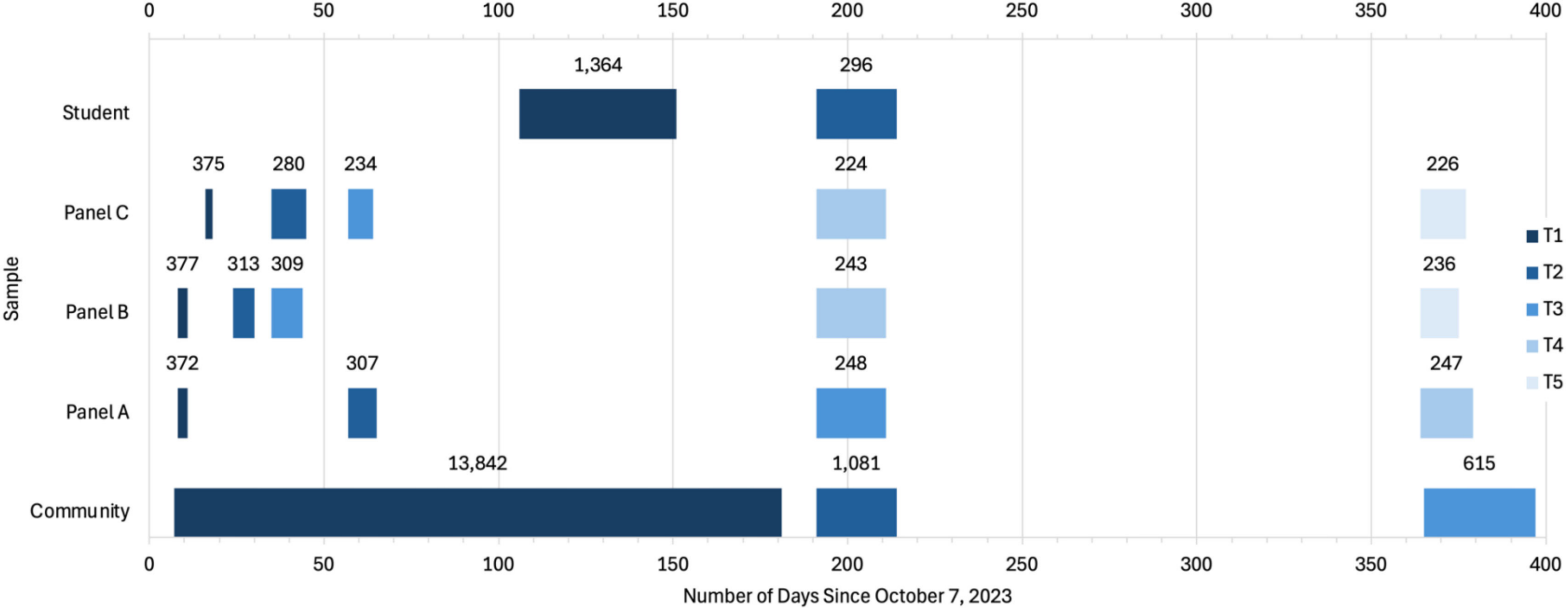
Data collection period and sample size according to sample wave. Bars and associated values represent the period of data collection and sample size, respectively, for each sample wave, with one exception. The Community Sample-T1 bar reflects the period during which participants were potentially eligible to be recruited for T2 (October 15, 2023, through April 5, 2024) rather than the entire study period (through May 1, 2024), whereas the sample size reflects the total number of participants from the entire study period. Specifically, 1,815 of the 13,842 participants in this wave were not eligible for recruitment to T2 because their T1 response was too recent (i.e., they responded between April 6-May 1, 2024, which is outside of the study period range indicated here). Otherwise, sample sizes include participants who were not considered for recruitment in subsequent waves (i.e., no valid contact details in the Community and Student Samples, or failed attention checks).

The six-month and one-year follow-up points were chosen because enough time passed to allow for the stabilization of (mal)adaptive reactions to the initial shock of war (23). Additionally, for this reason these are common benchmarks in studies of psychological adjustment after trauma exposure (24), thus enabling meaningful comparisons with existing literature. The project may develop further as we add data from future research. See **Figure 2** for the study periods and sample sizes according to sample wave thus far.

##### Consent, compensation, and sample characteristics

Participants in all samples provided renewed consent at each time point before completing the relevant assessments. Upon survey completion, participants were debriefed and provided with contact information for psychological support should they require assistance. Panel participants at all waves were compensated by the panel company for their participation, and 250 NIS vouchers were raffled off to those who participated at the six-month and one-year follow-ups. Participants in both the Community and Student Samples were offered to enter a raffle for 250 NIS vouchers in return for their participation. At the time of the raffles, 250 NIS was worth approximately 67–69 US dollars. See **Table 1** for a summary of demographic characteristics (gender, age, religiosity, education, and relative income) for each sample, and **Supplementary Table S2** for the same statistics according to sample wave.

**Table 1:** Sample sizes and demographic statistics according to sample.

Variable/level	Panel sample			Community sample	Student sample
	Panel A	Panel B	Panel C		
Sample size	372	377	375	13,842	1,364
Gender					
Male	48.1%	46.7%	48.8%	42.2%	37.0%
Female	51.3%	53.3%	50.9%	57.3%	62.8%
Other	0.5%	0%	0.3%	0.4%	0.3%
Missing	0%	0%	0%	0.1%	0%
Age (years)					
Mean (SD)	43.4 (15.5)	44.0 (16.2)	43.8 (16.4)	43.9 (15.3)	27.2 (6.3)
Median [Min, Max]	42 [18, 83]	41 [18, 81]	43 [18, 85]	43 [18, 100]	26 [18, 70]
Missing	0.5%	0.3%	0.3%	0.5%	0.1%
Religiosity					
Secular	44.9%	41.4%	40.3%	55.4%	51.1%
Traditional	35.8%	36.6%	36.8%	20.6%	17.0%
Religious	12.6%	11.9%	12.5%	13.3%	24.7%
Ultra-Orthodox	6.2%	8.8%	9.1%	6.0%	2.4%
Other	0.5%	1.3%	1.3%	4.4%	4.6%
Missing	0%	0%	0%	0.3%	0.1%
Education^a^					
Elementary school	1.1%	0%	0.8%	1.1%	
High school	21.8%	24.1%	21.3%	16.2%	
Post-secondary education^b^	25.8%	22.0%	26.1%	17.4%	
BA	29.0%	34.7%	36.0%	32.5%	
MA	18.8%	15.4%	12.8%	24.9%	
PhD	2.4%	1.6%	0.8%	4.7%	
Higher religious studies	1.1%	1.9%	1.9%	2.8%	
Missing	0%	0.3%	0.3%	0.4%	
Relative income					
Much lower than average	27.7%	29.4%	33.6%	27.6%	70.8%
Lower than average	17.7%	21.2%	16.8%	13.8%	10.0%
Average	26.3%	29.7%	26.9%	20.1%	8.8%
Higher than average	20.2%	14.9%	16.8%	22.6%	7.5%
Much higher than average	7.8%	4.2%	5.3%	14.6%	2.3%
Missing	0.3%	0.5%	0.5%	1.3%	0.7%

All statistics reflect demographic characteristics of all participants (even those not eligible for subsequent recruitment, e.g., failed attention check) in the samples at T1.

^a^ At T1 of the Student Sample, participants reported which of the following degrees they were working toward: BA (64.3%), MA (22.4%), PhD (7.4%), or medical school (5.5%), with 0.4% missing values.

^b^ This category represents any post-secondary education that is not otherwise specified (e.g., non-academic degrees, partial tertiary education).

### Materials

Participants in all samples completed a battery of assessments related to psychological function, risk, resilience, and the ongoing conflict. All study samples included assessments of core constructs from each of these main study categories. However, to maintain reasonable survey length, we rotated most measures between samples and/or waves. In addition, some measures were only included for part of the data collection duration (especially in the case of the Community Sample-T1, the duration of which exceeded 6 months). Finally, we randomized the presentation of some non-core measures between participants. All materials were presented in Hebrew. The following is a brief outline of select measures from each category. See **Table 2** for a comprehensive list of constructs assessed in each sample (see **Supplementary Table S3** for a comprehensive list of constructs, measures, and items according to sample wave, as well as multi-item measure Cronbach alphas).

**Table 2:** Study constructs according to sample.

Category and construct	Panel sample			Community sample	Student sample
	Panel A	Panel B	Panel C		
Psychological function					
Anxiety & depression (22, 25–27)	X	X	X	X	X
Anxiety & depression symptoms (49)	X	X		X	
Positive & negative affect (28, 29)	X	X	X	X	X
General well-being (49)	X	X	X	X	
Life satisfaction (74)	L	L	L	L	L
Post-traumatic stress disorder (75, 76)	L	L	L	L	L
Physical & emotional pain (30)				X	X
Prosocial behavior (49)	X	X	X	X	X
Psychological risk and resilience					
Values (31)	X	X	X	X	X
Religiosity & spirituality (49, 77)	X	X	X	X	X
Religious & spiritual change	L	L	L	X	X
Religious coping (78, 79)	L	L	L	L	L
Primal world beliefs (56)	C	C	L	L	L
Emotion regulation (80)	C	C		C	
Heroism (81)	X	X		X	
Self-criticism (82)	X	X	X	X	
Self-efficacy (36)		L	X		
Personality traits (32)	L	L	L	R1	R1
Attachment (83)	L	L	L	L	L
Psychological closeness to others (84)	L				
Interpersonal trust (40)	X	X	X	X	X
Loneliness (85)	L		L	X	X
Trait empathy (33)	L	L	L	R2	R2
Growth mindset (86, 87)	L	L	X	L	L
Post-traumatic growth (34, 35)	L	L	L	L	X
Meaning (37)	L	L	X		
Optimism (88)			L		
Optimism about Israel	L		L	X	X
Emotional closeness to Israel (40)	X	X	X	X	X
Israeli identity (38, 39)	X	X	X	X	
Jewish identity (39)	L				
Trust in local authorities (41)	X	X	R2	X	
Trust in institutions (40, 41)	X	X	R2	X	
Situational risk and resilience					
Received empathy	L	L	L	R2	R2
Family function (52)		L			
Social support (51)	X	X	X	X	X
Community relationships (49)	X	X	X	X	X
Community resilience (50)	X	X	R1	X	
National resilience (53)	X	X	R1	X	
Exposure to terror & war (42, 43)	X	X	X	X	X
Military service	X	X	X	X	X
Bomb shelter access	X	X	X	X	X
News consumption (40, 47, 48)	X	X	X	X	X
Financial impact (85)	X	X	X	X	X
Displacement	L	L	L	C	C
Perception of current situations (89)			X		
Disability of self or close others (44–46)				C	
Public discourse					
Criticism versus unity	C	C	R2		
Role of government vs citizens (54)	C	C			
National priorities of war		L			
Israel-Hamas negotiations (20, 55)	L	L	L	X	
Opinion concerning Hezbollah				X	
Opinion concerning Iran	L	L	L	X	L
General					
Open-ended questions	X	X	X	X	X
Demographics	X	X	X	X	X
Attention checks	X	X	X	X	X

X, C, and R, indicate inclusion of the construct (partial or complete) in at least T1 in the relevant sample, as follows. X indicates that the construct (partial or complete) was included for all participants. C indicates that questions in the relevant section were conditionally displayed based on participant responses to a filter question. R indicates that questions in the relevant section were randomized across participants. For example, in the Community Sample, the personality trait measure (R1) was randomized against measures of trait and received empathy (R2) so that approximately half of the participants received either the personality trait measure or the empathy measures, but not both. L indicates inclusion of the construct (partial or complete, and either for all participants, conditional, or randomized) in at least 1 later timepoint, but not at T1, in the relevant sample. See **Supplementary Table S3** for a more detailed description of measurement tools and items used across all waves and for measure/item rotation within each wave (e.g., measures that were included for only part of a data collection period).

#### Psychological function

Participants completed various measures of anxiety and depression. In all samples and waves, the PHQ-4 (22) was administered. At some of the waves, we administered more elaborate versions of this questionnaire—the General Anxiety Disorder-7 (GAD-7) (25) and PHQ-9 (26)—and/or the Overall Anxiety Severity and Impairment Scale (OASIS) (27). As another example, participants rated the extent of their positive and negative emotions during the previous week (28, 29), and their physical and emotional pain during the previous two weeks (30).

#### Psychological risk and resilience

To assess psychological risk and resilience, we administered measures of various personality-related constructs, such as values (31), traits (32), empathy (33), post-traumatic growth (34, 35), self-efficacy (36), and meaning (37). At another level, we assessed participants’ connection to their broader social and cultural context via measures relating to collective identity (both Israeli and Jewish) (38, 39), trust in institutions (40, 41) and national attachment and optimism (40).

#### Situational risk and resilience

To assess situational risk and resilience, participants reported on their direct and indirect exposure to adverse conditions and stressors and their exposure to protective factors. For example, in addition to a general measure of war exposure (adapted from previous research on exposure to terror in Israel) (42, 43), participants completed assessments of various other relevant experiences and circumstances, such as displacement from one’s home, disabilities of the self and close others (44–46), news consumption patterns (40, 47, 48), and impact of the war on personal finances. Additionally, we administered measures assessing both proximal and distal support systems. For example, participants completed measures of received empathy, and of social support and cohesion at the personal, familial, communal, and societal/national levels (40, 49–53).

#### Ongoing conflict

Finally, we asked participants about several other issues relevant to their experiences during the war (e.g., what is most important to them, what they are most worried about, if the war has changed them, what helps them cope) in an open-ended question format interspersed throughout each survey, in addition to a demographic assessment.

**Figure 3:**
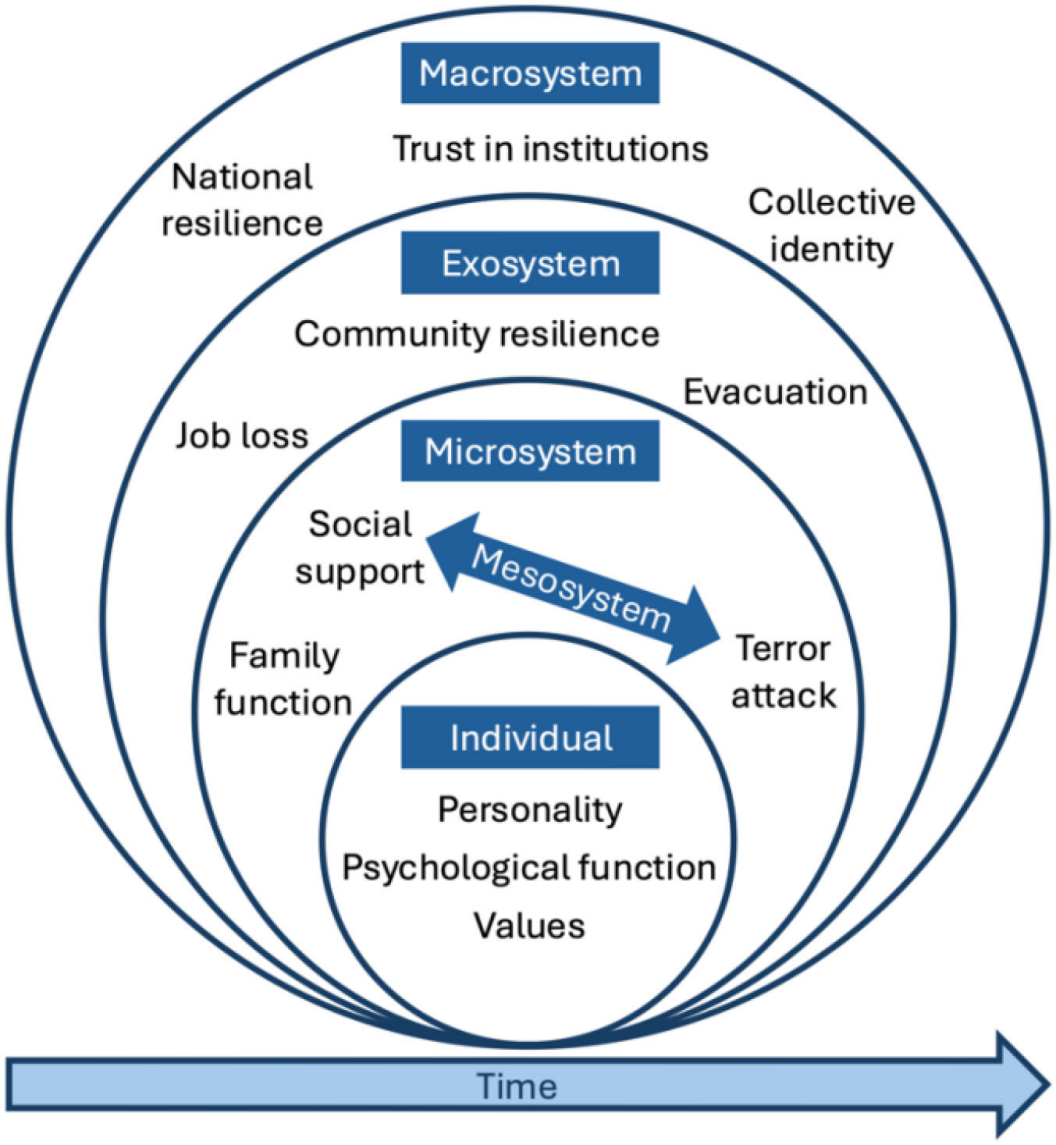
Illustrative organization of variables within a bioecological framework.

When relevant, we considered additional issues concerning the ongoing conflict and public discourse, such as attitudes toward the role of the state versus civil society in providing for the citizens’ needs (54) and the Israel-Hamas hostage negotiations (20, 55). Many significant events occurred throughout the duration of the war and data collection periods. For example, the Israeli ground incursion into Gaza began on October 27, 2023 (day 21), a temporary cease-fire and hostage deal began on November 24, 2023 (day 49), and an unprecedented airstrike from Iranian soil occurred on April 13, 2024 (day 190). Delineating events that may be psychologically relevant to the current samples is beyond the scope of this paper. We are planning to add to our database additional external data (e.g., concerning air raid sirens, rocket fire, bombings, civilian casualties, political events, protests, assassinations, etc.) to indicate the timeline, frequency, and location of relevant events. See **Figure 3** for an example organization of variables within Bronfenbrenner’s bioecological perspective.

### Preliminary results and discussion

Given the vast array of both quantitative and qualitative data collected in longitudinal designs, there is no one set of relevant hypotheses, nor one appropriate analytical approach that should be employed to examine the effects of CTS on individuals and groups. Therefore, to illustrate the potential of this project we present here select preliminary findings of varying natures. In this way, readers can gain insight into some of the types of questions that will be possible to address with our project. We begin with some descriptive statistics concerning exposure to the effects of war (e.g., rocket attacks, financial hardships) and a potential protective factor that is influenced by the war context (i.e., trust in institutions). We then focus on individuals’ adjustment and the unfolding of war itself by demonstrating how personal values relate to internalizing symptoms, coping mechanisms, and opinions about how the war should progress. The aim is for this brief sampling of findings to stimulate a broad set of research questions and the development of more complex models that consider at once the interaction between multiple variables across time. In the discussion section, we elaborate on more advanced analytical approaches that our project enables, such as natural language processing and machine learning.

#### Exposure to the effects of war

**Figure 4:**
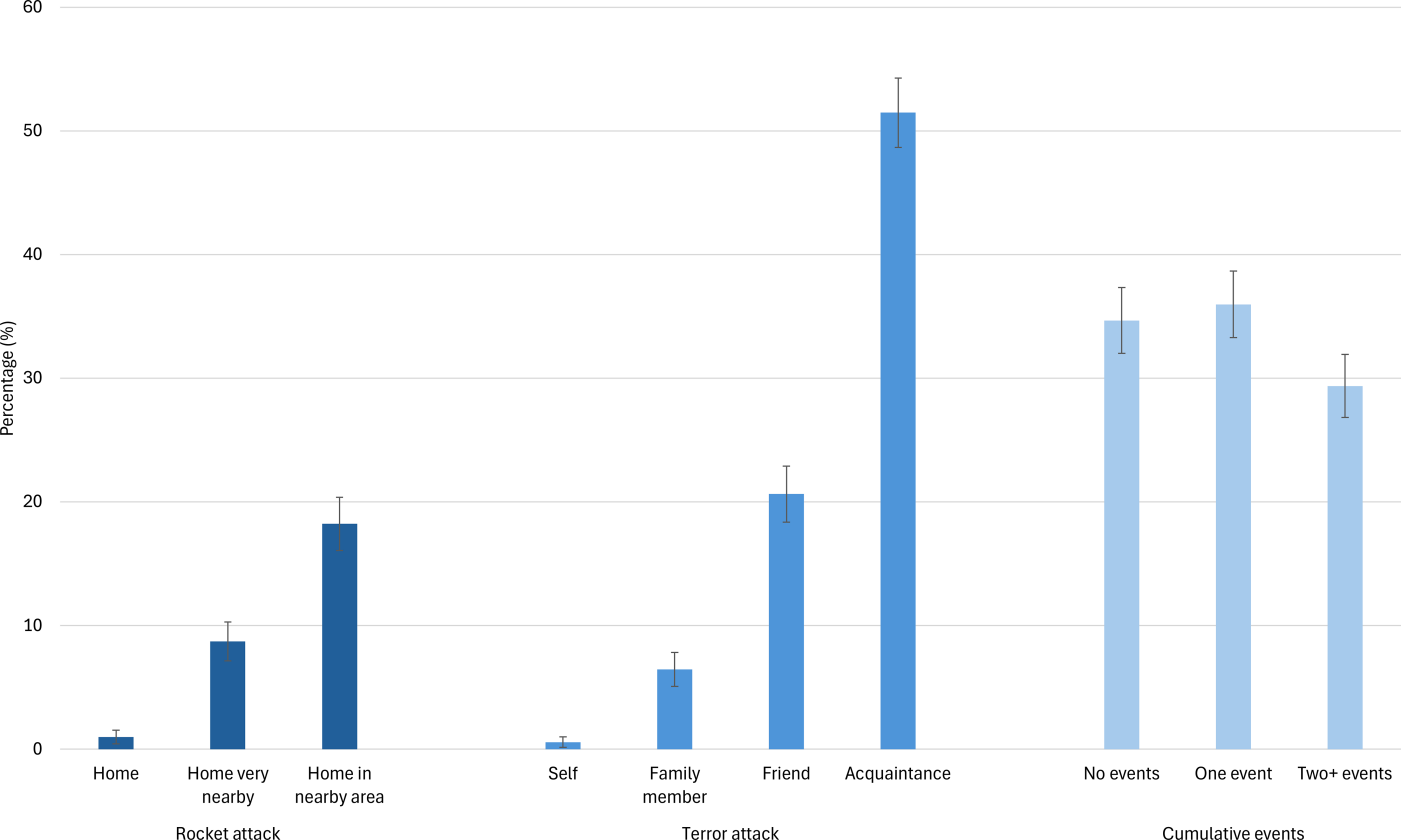
Participants’ exposure to specific war events and cumulative levels. Error bars are 95% confidence intervals.

Effectively studying psychological function during war requires attention to the effects of both direct and indirect exposure to the events of war. The following are select findings from the Student Sample (data collected between January 22 and March 6, 2024) concerning the levels of exposure to two major types of events during the current war—terror attacks and rocket attacks. Specifically, we asked participants whether (a) they were injured in an attack, or whether close others (i.e., family members, friends, acquaintances) were injured or killed (4 items), and (b) their house, a house located very close to theirs, or a house located in a nearby area were hit by rockets (3 items) (42, 43). Exposure rates vary by type and proximity (**Figure 4**). For example, 20.6% of the participants reported having a friend who was injured or killed in a terror attack, and 8.7% reported living very close to a house that was hit by a rocket. There was also substantial variance in participants’ degree of overall exposure, whereby 34.7% reported not experiencing any of the events, 36% reported experiencing one event, and the remaining 29.4% reported experiencing two or more events. Actual exposure rates in the population may have been even higher, as those who were more substantially affected were likely less available to participate in the study. Nevertheless, variability in exposure indicates that our research is well-positioned to study the direct and indirect effects of war on individuals, while considering the type, proximity, and extent of exposure. These data will be informative of the environment in which the participants were living, and subsequent findings should be viewed in context of these significant and potentially traumatic events.

Although much of our project aims to elucidate how psychological adjustment is affected by direct and indirect exposure to the terror attacks and fighting (e.g., being displaced or having a family member hurt in the fighting), there are additional, more distal, effects of war that should be considered. One direction is the detrimental effect war has on people’s livelihood and economic stability, and the association between economic events and internalizing problems (anxiety and depression). Individuals were asked about six events that could have potentially hurt them economically (e.g., harm to their business, losing their jobs). Early in the war (November 2023), only 23.7% of participants in the Panel Sample were affected by at least one event. Within a few weeks (December 2023), 37.5% had encountered one event and 8.9% two events or more (*χ^2^*(*df* = 6) = 64.21, *p* <.001, for prevalence across time). The number of individuals exposed to at least one of the events remained stable (April 2024: 33.1%, and a year into the war, October 2024: 33%). Percentages are not fully comparable across time points due to recruitment criteria to subsequent waves and sample composition (see Method section). These findings suggest that to the horrors of exposure to death and destruction in the war, seemingly mundane economic concerns are added, which may themselves take a toll on mental health. We plan to investigate the effect of such events on anxiety and depression across time.

#### Trust in institutions

**Figure 5:**
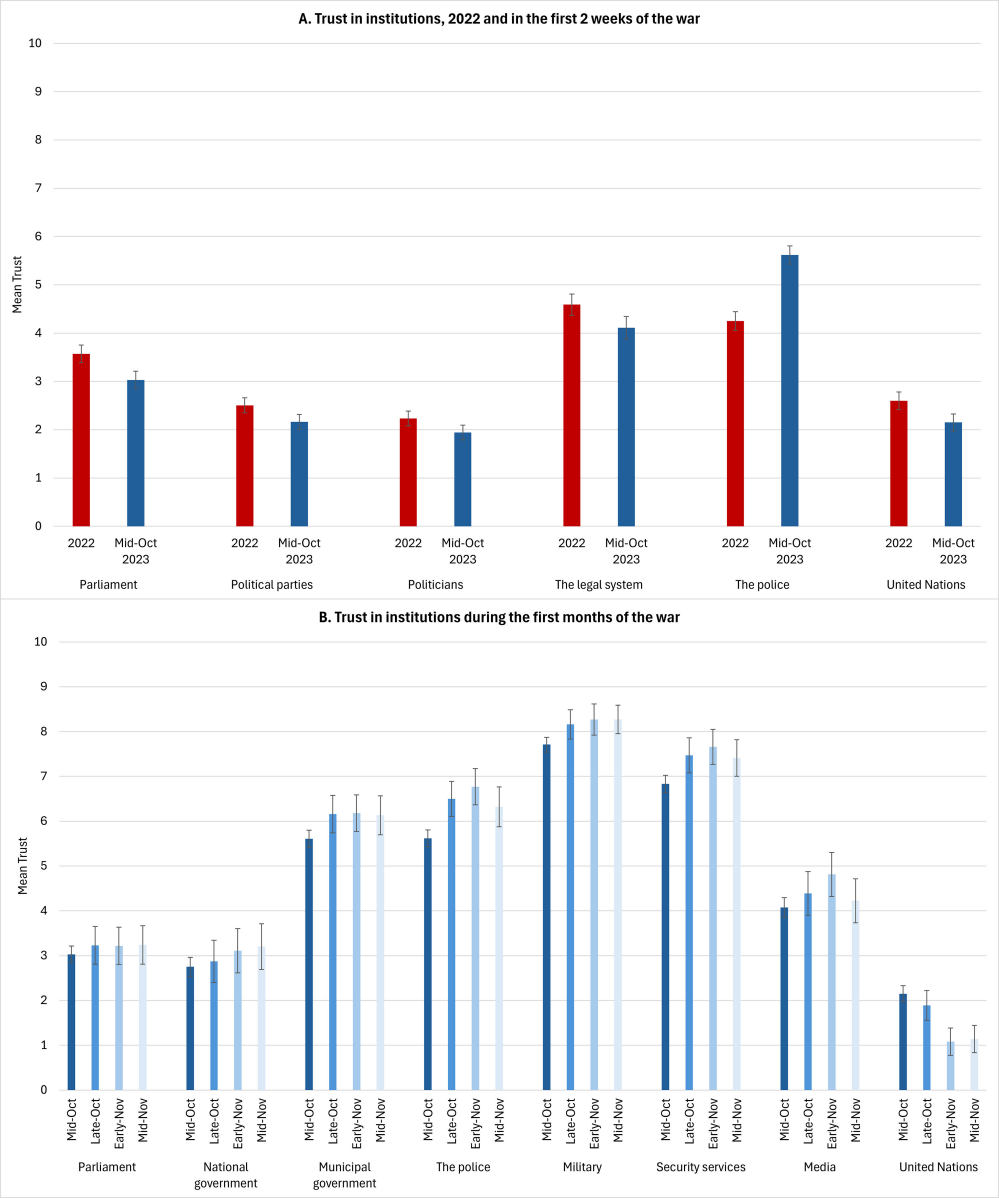
Trust in institutions **(A)** pre-war and in the first two weeks of war, and **(B)** during the first months of war. 2022 comparison data are from the publicly available data of the 10^th^ round of the European Social Survey (https://www.europeansocialsurvey.org/) (40), a demographically representative sample of *N* = 844 individuals from the Jewish-Israeli majority. Number of participants was 731 for mid-October data collection (Panels A and B at T1), and 167 for the late-October data collection (subsample of Panel C at T1). Data from early-November (*N* = 150) and mid-November (*N* = 147) are from subsamples of the mid-October sample (Panel B at T2 and Panel B at T3, respectively). Subsamples were used because these items were randomly presented to approximately half of these samples. Error bars are 95% confidence intervals.

The surprise by which the October 7 attacks took Israeli society may have been an important stressor affecting population adjustment. Overall levels of trust in political institutions in Israel were very low already in 2022 (40) (during a political stalemate that preceded the 2023 judicial reform crisis). In our Panel Sample, they were even lower in mid-October, shortly after the beginning of the war (**Figure 5a**). Interestingly, levels of trust in political institutions increased slightly within a few weeks. At the same time, trust in the United Nations, which was already very low in 2022, further decreased as the war proceeded. Despite their failure to prevent the October 7 attacks, trust in security forces was high at the beginning of the war, and further improved over time (**Figure 5b**). A future step is to see the role of trust in institutions, and the improvement thereof, in mental health.

#### Meaning-making and war adjustment

To better understand individuals’ adjustment during war, we aimed to explore how people’s thoughts and beliefs related to their coping mechanisms and how they perceived war-related issues. This included examining participants’ primal world beliefs (e.g., perceiving the world as good or safe) (56) and their optimism regarding the country’s future. Values—desirable, abstract goals that vary in importance across individuals and cultures and guide personal behavior and the evaluation of others (14)—play a significant role in shaping how people interpret events and form opinions (57, 58). Therefore, relying on Schwartz’s (14) theory of human values and the 10 core values it identified, we highlight the role of values in individuals’ perceptions and attitudes toward war and in their coping.

**Figure 6:**
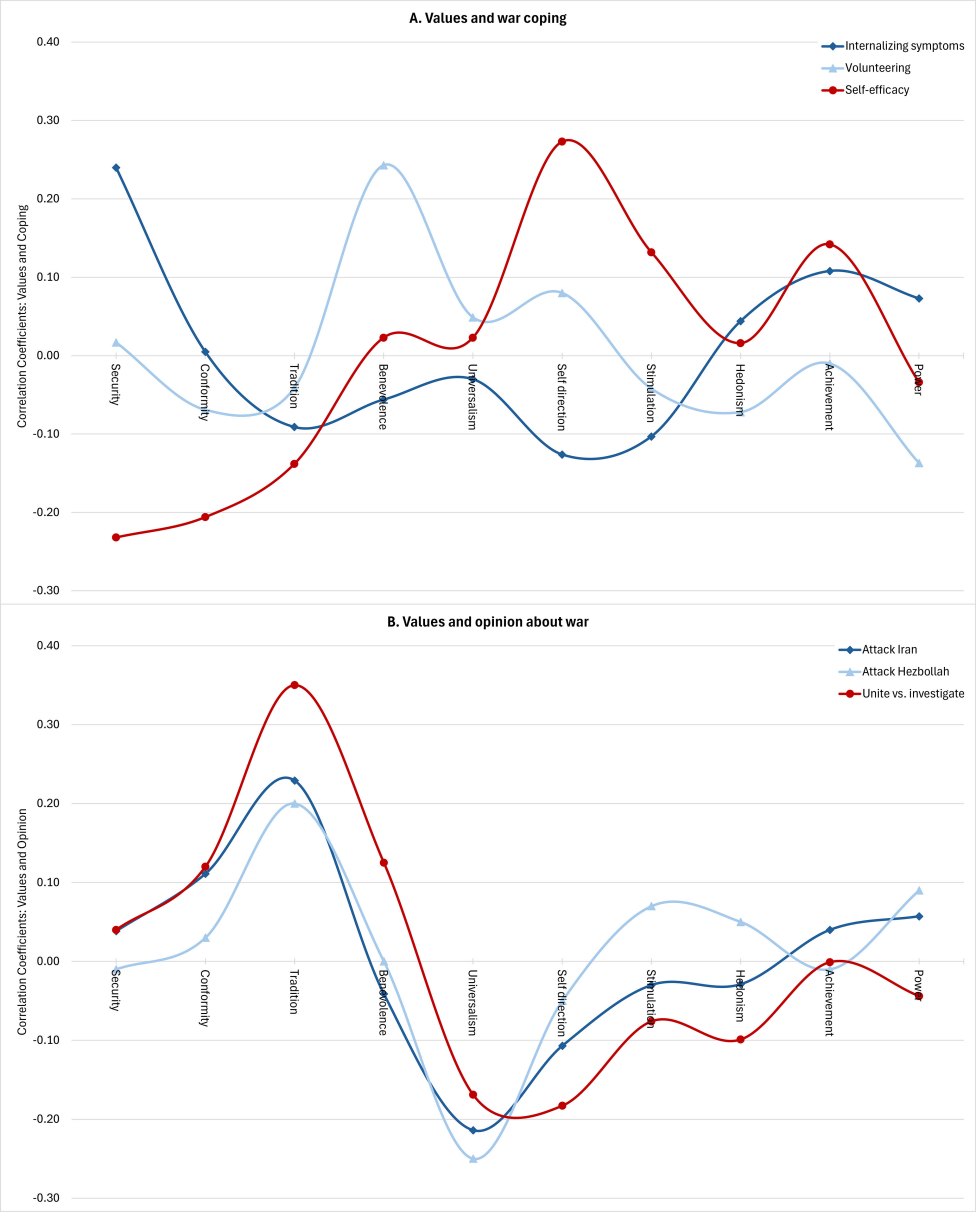
Correlations between personal values and **(A)** coping during war and **(B)** opinions about war. Correlations for internalizing symptoms and volunteering (*N* = 1,079), self-efficacy (*N* = 352), and unite vs. investigate (*N* = 762) are based on Panel Sample data from October 2023 (Wave 1). Coefficients regarding attacking Iran are based on Panel Sample data from April 2024 (*N* = 667, six-month follow-up), and coefficients regarding attacking Hezbollah are based on Community Sample data from February-April 2024 (*N* = 3,857). Coefficients for Iran and Hezbollah reflect Spearman correlations between each value and an ordinal opinion scale where 1 = No, 2 = Undecided, 3 = Yes.

The relationship between values and mental health is complex, as there is limited evidence supporting a “healthy values” approach where specific values directly correlate with well-being (59, 60). Instead, research typically indicates that the alignment between a person’s values and their environment positively influences well-being (61). People who manage to fulfill their values have been shown to have better well-being (60). In the context of war, we found a positive correlation between internalizing symptoms and an emphasis on security values, suggesting that in highly threatening situations, in which security is hard to achieve, individuals who prioritize this value may experience greater distress (**Figure 6a**).

Two important but distinct coping mechanisms related to mental health in stressful situations include self-efficacy and prosocial behavior (62, 63). Self-efficacy, the belief in one’s personal ability to manage in situations (64), was positively associated with self-direction values (autonomous thought and action) and negatively associated with conformity values (adhering to social norms, rules, and regulations) and security values (focused on minimizing threats to oneself and society). During the war, volunteering became a vital coping strategy, with nearly 50% of Panel Sample participants reporting volunteering within the first month. Benevolence values (emphasizing the welfare of close others) were positively related to volunteering, while power values (emphasizing resource acquisition, social influence, and dominance) were negatively associated with volunteering during the conflict (**Figure 6a**). Thus, different values may relate to using different coping mechanisms, that can be either self-focused or other-focused.

Furthermore, power values have been linked to support for military action in conflicts between nations, whereas universalism values (promoting open-mindedness, tolerance, and caring for all humans) have been linked to supporting peaceful resolutions (65, 66). Thus, values relate to how individuals form opinions about war developments and the leadership’s decisions regarding subsequent steps.

For example, as Israel entered the war amidst a severe internal political crisis, coupled with revelations of military and political leadership failures following the October 7 attacks, public debate arose regarding the appropriateness of investigating the state’s failure during the conflict. Values of universalism and self-direction were associated with support for an immediate investigation, while conformity, benevolence, and particularly tradition (commitment to cultural or religious heritage)—all values that emphasize ingroup cohesion—were linked to opposing the investigation and advocating for unity against Hamas.

Amid ongoing tensions at Israel’s northern border, including rocket attacks from Lebanon, we asked participants: “Should a war against Hezbollah be initiated?” Similarly, after the Iranian attack on Israel on April 13-14, 2024, involving hundreds of missiles and drones, we asked: “Do you think Israel should retaliate against Iran?” In both scenarios, universalism values were linked to opposing military action, while tradition values were associated with supporting it (**Figure 6b**). These findings suggest that values shape how individuals perceive or form opinions about war, which may have implications for future stress and coping.

## Discussion

This work presents the first year in an expansive ongoing longitudinal study conducted over the first years of the Hamas-Israel war. It includes three different study samples, each proposing unique advantages, that together can provide a comprehensive outlook on psychological risk and resilience during war. First, the Panel Sample is a representative sample of the Jewish adult population in Israel, enabling investigation of phenomena of interest at the level of the general public. Second, the Community Sample is a very large sample of diverse participants (from which representative samples can be drawn) across more junctures of the war—especially during the first 6 months. Third, the Student Sample may facilitate the study of young adults’ experiences during the war. This group was disproportionately affected in the October 7 attacks and may be highly exposed to casualties among close friends and siblings, making it an important group to study (67). Together, these samples provide powerful sources for replication and allow for a broad examination of the Jewish Israeli public’s experiences during the war. The multivariate, longitudinal nature of all study samples offers a unique opportunity to comprehensively examine the individual, community, and societal effects of continuous mass trauma over time.

Beyond the first year described in this paper, we continue study planning and data collection in different subsamples. For example, a hostage deal was carried out during a temporary ceasefire in January 2025. During the ceasefire, Panel Sample participants completed an abbreviated survey centered on assessing their views of the deal (along with some psychological well-being indicators and religion and demographic variables) that is not part of the current report. In addition, a two-year follow-up is planned for the Panel Sample. We will keep on updating the project based on funds and interest from the scientific community and practitioners.

Preliminary analyses presented in the current work provide a glimpse into the varied effects of continuous trauma on psychological factors of affected populations. We presented select findings on risk (e.g., exposure to adverse events), resilience (e.g., trust in institutions), and psychological function (e.g., internalizing, opinions about war) from the different samples. The findings indicate that exposure to war-related risks is widespread, as is exposure to adverse events such as financial troubles due to war, which may affect depression and anxiety levels. Similarly, psychological well-being may be supported by individuals’ trust in institutions, which varied across institutions and over time, possibly serving as a protective factor in the context of war related stresses. Finally, we also demonstrated the important role of personal values in predicting internalizing symptoms, coping mechanisms, and opinions regarding the continuation of the war. These findings point to concerns regarding important social issues such as low levels of trust in the government, economic hardships, and narratives concerning the unfolding of war. Together these findings are a partial illustration of the potential power of this study and of the research questions that can be answered with it. Indeed, initial findings from this project are at various stages of completion at the time of this writing, including studies of: (a) the contribution of values to opinions regarding the Israel-Hamas negotiations (20) (b) religious and spiritual changes due to war (19), and (c) distress trajectories in the first 6 months of the war and their contribution to PTSD risk (68).

Our ultimate goal is to publish the data. Compared to larger regions, in the small Israel society there are risks of violating privacy because it may be relatively less difficult to identify people (69). Such risks can be minimized with techniques such bucketing (e.g., recoding age in years to grouped) or by removing demographic data, although this reduces the ability to study relevant variables. Publishing data from an ongoing study may result in papers that will rely on incomplete data; at the same time, it enables more substantial investigations to be conducted at later time points. However, we aim to collect data that will enable the study of the long-term effects of war. Therefore, we present the protocol now and call for collaborations that could result in meaningful findings that contribute to psychological science now and can guide further investigations of developing data or even future data collections.

While our research focused on the October 7 events in Israel and the ensuant Hamas-Israel war, its findings might be relevant to other contexts of exposure to war and trauma. Occurrences of CTS that characterize populations exposed to war are disturbingly frequent across the world. Moreover, research has shown considerable differences between acute trauma and continuous trauma, with a strong inclination in existing research to focus on the impact of acute trauma. Therefore, our project is positioned to make important contributions to characterizing psychological responses in large populations exposed to continuous trauma, and to studying intra- and inter-personal factors that contribute to risk and resilience.

Although this study has several strengths, such as multivariate, mixed-method, longitudinal designs in multiple samples covering wide portions of the population, it also has limitations. First, recent research suggests that CTS contexts may result in some symptoms that are distinct from traditional assessments of PTSD (70). We included well-being assessments (e.g., PHQ-4) at the outset of an evolving war, before we knew it would present a CTS context, and maintained these measures for consistency. Therefore, our measures of mental health functioning might not capture the full gamut of potential CTS-related symptoms. Nevertheless, investigations across multiple measurement times (especially in the Panel Sample) may indicate that participants are experiencing continuous stress. Second, our study relied entirely on self-reports. To address this, we plan to integrate our data with other data such as official war exposure rates based on geographic location. Additionally, relying on the fact that we employed longitudinal designs, we can reach out to participants again to obtain additional data from other reporters (e.g., partner reports on adjustment). Third, despite substantial efforts, we were unable to reach enough participants from the Arab population in Israel (the sample of Arab participants was very small; see Method section). Thus, our samples do not fully represent the entire population in Israel. The experiences of Arab and Palestinian citizens of Israel might differ substantially from the experiences of Jewish-Israelis due to the added complexity of their minority status and Palestinian identity. Research concerning the experiences of Arab-Israelis during such nation-wide trauma exposure is needed to complement our research program. Similarly, the consequences of war for those in bordering areas, particularly in Gaza, have been widespread and severe, resulting in mass casualties, displacement of civilians, and ongoing suffering and trauma. Finally, our focus on only one war may limit our ability to generalize findings. It is important to study all affected populations in the current war as well as in other conflicts. We therefore aim to share our methods with researchers in other regions and warzones, and it is our hope that this work may create opportunities for cross-border collaborations in the future.

The main purpose of this report is to present our research endeavors to the scientific community. We are hoping for local and international collaborations with researchers from various fields beyond psychology and mental health. The breadth of our data collection will result in an unprecedented database in the context of continuous trauma that will hold immense potential for scholars from various disciplines, from political science and history to economics and public policy. For example, we note the cultural and historical importance of the text responses collected by thousands of participants in the study. We believe that these will reflect a valuable historical record for the examination of the general public’s opinions and sentiments during the war. Moreover, utilizing advanced methodologies to examine such extensive volumes of language-based data might provide a unique opportunity for incorporating natural language processing (NLP) methodologies, which have been increasingly used in mental health research (71).

Another potential avenue for research involves utilization of machine learning methodologies. As mentioned, in the case of continuous trauma, various factors are expected to influence risk and adjustment, and complex processes may be characterized by non-linear relationships that traditional statistical methods might overlook (72). This has led to increased utilization of machine learning methodologies in studying psychological adjustment following trauma, specifically post-traumatic stress disorder. Machine learning procedures can process large volumes of multivariate data, enabling to develop sophisticated data models and discover intricate patterns and interactions among various risk and resilience factors across different levels and timepoints (73). The wide-ranging nature of the variables assessed in sizeable samples will provide a unique opportunity to utilize machine learning models to predict psychopathology (or other outcomes) during continuous trauma.

In conclusion, this study presents a meaningful resource for understanding the psychological, social, and community-level impacts of continuous trauma. Its breadth and longitudinal, multivariate design provide ample opportunity for studying a diverse set of research questions and may serve as a valuable foundation for future research, which may draw on its procedures for designing studies of CTS in other contexts. We hope that the dataset and findings generated by this work will not only deepen the understanding of trauma responses but also inspire collaborations across disciplines and contribute to informed policies and interventions to support populations affected by continuous mass trauma.
