# Supplementary material for: Psychological function in the context of protracted stress during war: a multi-sample, multivariate longitudinal study
Source: Front Psychiatry. 2026 Apr 23;16:1729795. doi: 10.3389/fpsyt.2025.1729795 (PMC13149444; doi:10.3389/fpsyt.2025.1729795)
Supplement: Supplementary file 1 [file DataSheet1.pdf]

Table of Contents

Table S1 2
Table S2 3
Table S3 4
Additional Method Details 8
Community and Student Sample recruitment 8
Participation inclusion and data retention 8
References 9

Table S1

Representativeness of the Panel Sample

	General population	Panel sample
**Gender**		
Male	48.7%	48.1%
Female	51.3%	51.9%
**Age (years)**		
20-44	48.9%	52.7%
45-64	30.6%	34.2%
65 and above	20.5%	13.1%
**Religiosity**		
Secular	43.9%	41.7%
Traditional	32.9%	36.4%
Religious	11.5%	12.3%
Ultra-Orthodox	10.9%	8.4%
Other	0.0%	1.1%
Unknown/missing	0.7%	0.0%

Note. The general population statistics for gender, age, and religiosity describe the Jewish population in Israel aged 20 and above, as The Central Bureau of Statistics[1] does not provide the relevant data on individuals below this age group. For comparison, the Panel Sample statistics describe all participants at T1 who identified as Jewish, were age 20 or above, and did not fail any attention checks (93.1% of the entire sample; n = 1,047). Religiosity percentages do not sum to 100 due to rounding.

Table S2
Sample Sizes and Demographic Statistics According to Sample Wave

	Panel Sample									Community Sample			Student Sample	
	Panel A			Panel B			Panel C							
	T1	T3	T4	T1	T4	T5	T1	T4	T5	T1	T2	T3	T1	T2
**Sample size**	372	248	247	377	243	236	375	224	226	13,842	1,081	615	1,364	296
**Gender**														
Male	48.1%	50.4%	48.6%	46.7%	51.0%	50.4%	48.8%	49.6%	50.4%	42.2%	32.3%	33.5%	37.0%	37.5%
Female	51.3%	49.2%	51.4%	53.3%	49.0%	49.6%	50.9%	50.4%	49.6%	57.3%	67.5%	66.5%	62.8%	62.5%
Other	0.5%	0.4%	0%	0%	0%	0%	0.3%	0%	0%	0.4%	0.2%	0%	0.3%	0%
Missing	0%	0%	0%	0%	0%	0%	0%	0%	0%	0.1%	0%	0%	0%	0%
**Age (years)**														
Mean (SD)	43.4 (15.5)	46.7 (15.2)	47.0 (15.1)	44.0 (16.2)	48.0 (15.7)	47.9 (15.7)	43.8 (16.4)	47.0 (16.2)	46.7 (15.7)	43.9 (15.3)	44.6 (15.5)	47.5 (15.7)	27.2 (6.3)	27.2 (6.4)
Median [Min, Max]	42 [18, 83]	45 [18, 83]	46 [19, 83]	41 [18, 81]	49 [18, 79]	49 [20, 80]	43 [18, 85]	47 [19, 81]	46.5 [19, 81]	43 [18, 100]	43 [18, 89]	47 [18, 89]	26 [18, 70]	26 [19, 70]
Missing	0.5%	0%	0%	0.3%	0%	0%	0.3%	0.4%	0%	0.5%	0.2%	0%	0.1%	0%
**Religiosity**														
Secular	44.9%	48.0%	50.2%	41.4%	43.6%	40.7%	40.3%	42.9%	43.8%	55.4%	60.5%	63.9%	51.1%	55.7%
Traditional	35.8%	33.9%	29.1%	36.6%	36.2%	38.6%	36.8%	36.6%	34.1%	20.6%	12.7%	14.0%	17.0%	13.2%
Religious	12.6%	12.1%	14.2%	11.9%	11.1%	11.9%	12.5%	12.9%	12.8%	13.3%	14.6%	13.5%	24.7%	24.7%
Ultra-Orthodox	6.2%	6.0%	6.5%	8.8%	8.2%	8.1%	9.1%	7.6%	9.3%	6.0%	6.9%	5.4%	2.4%	2.4%
Other	0.5%	0%	0%	1.3%	0.8%	0.4%	1.3%	0%	0%	4.4%	5.3%	3.3%	4.6%	4.1%
Missing	0%	0%	0%	0%	0%	0.4%	0%	0%	0%	0.3%	0%	0%	0.1%	0%
**Education**[a]														
Elementary school	1.1%	0.8%	1.6%	0%	0.8%	0.4%	0.8%	0.4%	0%	1.1%	0.3%	0.5%		
High school	21.8%	20.2%	19.4%	24.1%	23.5%	25.8%	21.3%	22.8%	22.1%	16.2%	12.0%	11.9%		
Post-secondary education[b]	25.8%	21.0%	19.8%	22.0%	25.1%	20.8%	26.1%	25.9%	24.8%	17.4%	17.4%	14.5%		
BA	29.0%	30.2%	30.4%	34.7%	30.5%	31.8%	36.0%	33.5%	37.6%	32.5%	32.4%	32.0%		
MA	18.8%	22.6%	24.3%	15.4%	17.3%	18.6%	12.8%	15.2%	14.2%	24.9%	29.4%	33.0%		
PhD	2.4%	3.2%	3.2%	1.6%	2.1%	1.7%	0.8%	0.9%	0.9%	4.7%	5.7%	5.5%		
Higher religious studies	1.1%	2.0%	1.2%	1.9%	0.8%	0.8%	1.9%	0.9%	0%	2.8%	2.7%	2.6%		
Missing	0%	0%	0%	0.3%	0%	0%	0.3%	0.4%	0.4%	0.4%	0.1%	0%		
**Relative income**														
Much lower than average	27.7%	25.8%	25.5%	29.4%	25.1%	24.6%	33.6%	28.1%	28.3%	27.6%	29.7%	28.5%	70.8%	68.6%
Lower than average	17.7%	14.1%	16.6%	21.2%	23.0%	19.9%	16.8%	16.5%	16.8%	13.8%	14.8%	13.8%	10.0%	10.8%
Average	26.3%	31.5%	25.9%	29.7%	29.2%	33.9%	26.9%	29.9%	31.0%	20.1%	19.9%	20.8%	8.8%	8.8%
Higher than average	20.2%	19.4%	22.7%	14.9%	18.5%	17.4%	16.8%	17.9%	18.1%	22.6%	23.8%	21.6%	7.5%	9.5%
Much higher than average	7.8%	8.9%	9.3%	4.2%	4.1%	4.2%	5.3%	7.1%	5.8%	14.6%	11.3%	14.3%	2.3%	1.7%
Missing	0.3%	0.4%	0%	0.5%	0%	0%	0.5%	0.4%	0%	1.3%	0.6%	1.0%	0.7%	0.7%

Note. Demographic variables in the Panel Sample were assessed only at T1 and the six-month and one-year follow-ups (i.e., T3 and T4 of Panel A and T4 and T5 of Panels B and C). Percentages may not sum to 100 due to rounding.
[a] At T1 of the Student Sample, participants reported which of the following degrees they were working toward: BA (64.3%), MA (22.4%), PhD (7.4%), or medical school (5.5%), with 0.4% missing values.
[b] This category represents any post-secondary education that is not otherwise specified (e.g., non-academic degrees, partial tertiary education).

Table S3

Study Constructs and Measures According to Category and Sample Wave

Category and Construct	Measure/Source[a]	Panel Sample														Community Sample			Student Sample		Cronbach Alphas
		Panel A				Panel B					Panel C										
		T1	T2	T3	T4	T1	T2	T3	T4	T5	T1	T2	T3	T4	T5	T1	T2	T3	T1	T2	
**Psychological function**																					
Anxiety & depression	PHQ-4[2]	X				X	X				X	X	X			X			X		.68-.81
Anxiety & depression[b]	PHQ-9[3]*		X	X	X			X	X	X				X	X		X	X		X	.91
Anxiety & depression symptoms[c]	European Social Survey[4]	X				X										X					.90
Anxiety	OASIS[5]		X				X	X			X	X	X								.92
Anxiety	GAD-7[6]*			X	X				X	X				X	X		X	X		X	.93
Positive & negative affect[d]	Children's Worlds Project[7], Feldman-Barrett et al.[8]*	X	X	X	X	X	X	X	X	X	X	X	X	X	X	X	X	X	X	X	.42-.77
General well-being[c,e]	European Social Survey[4]	X	X			X	X	X			X	X	X			X					.77
Life satisfaction	SWB[9]*			X	X				R1					R1			R1			R1	.96
Post-traumatic stress disorder	PCL-5[10,11]			X	X				X	X				X	X		X	X		X	.96
Physical & emotional pain[f]	Gilam et al.[12,13]*															X			X		.91-.92
Prosocial behavior[g]	European Social Survey[4]*	X	X	X	X	X	X	X	X	X	X	X	X	X	X	X	X	X	X	X	
**Psychological risk and resilience**																					
Values[h]	PVQ[14]*	X	X	X	X	X	R2	R2	X	X	X	X	X	X	X	X	X	X	X	X	.41-.76
Religiosity & spirituality[i]	European Social Survey[4], Fetzer Institute[15]*	X	X	X	X	X		X	X	X	X	X	X	X	X	X	X	X	X	X	.84
Religious & spiritual change[j]			X	X	X			X	X	X		X	X	X	X	X	X	X	X	X	
Religious coping	RCOPE[16,17]*			X	X				X	X				X	X		X	X		X	.96
Primals world beliefs[k]	PI-18[18]	C		X	X	C			R1					R1			R1			R1	.75-.83
Emotion regulation[k,l]	Tamir et al.[19]*	C	C			C	C	C								C					.47-.90
Heroism[m]	SHERS[20]*	X				X										X					.65
Self-criticism[m]	DEQ-A[21]*	X				X					X					X					.70
Self-efficacy	GSE[22]						R1	R1			X	X	X								.91
Personality traits[n]	BFI-2-XS[23]		X	X	X				X	X				X	X	R1	X	X	R1	X	.32-.62
Attachment	ECR-S[24]			X	X				R2				X	R2			R2			R2	.77-.82
Psychological closeness to others	IOS[25]*		X	X	X																.82
Interpersonal trust	European Social Survey[26]	X	X			X	X	X			X	X	X			X			X		.77
Loneliness[o]	Israel Central Bureau of Statistics [27]		X										X			X			X		
Trait empathy[n]	IRI[28]*		X	X	X				X	X				X	X	R2	X	X	R2	X	.39-.58
Growth mindset	Levontin et al. [29,30]*		X	X	X			X	X	X	X	X	X	X	X		X	X		X	.82
Post-traumatic growth	PTGI-SF[31,32]		X	X	X				R2	X		X	X	R2	X		R2	X	X	R2	.91
Meaning[p]	MIL[33]		X					X			X	X	X	X	X						.92
Optimism	LOT-R[34]*												X	X	X						.80
Optimism about Israel[q]			X										X			X			X		
Emotional closeness to Israel	European Social Survey[26]	X	X	X	X	X	X	X	X	X	X	X	X	X	X	X	X	X	X	X	
Israeli identity[c]	Roccas et al.[35]*	X	X			X					X					X					.90
Israeli identity	EIS [36]*		X	X	X																.79
Jewish identity	EIS[36] *		X	X	X																.81
Trust in local authorities[r]	The Israel Democracy Institute[37]	X				X	R2	R2			R2					X					

Category and Construct	Measure/Source[a]	Panel Sample														Community Sample			Student Sample		Cronbach Alphas
		Panel A				Panel B					Panel C										
		T1	T2	T3	T4	T1	T2	T3	T4	T5	T1	T2	T3	T4	T5	T1	T2	T3	T1	T2	
Trust in institutions[s]	The Israel Democracy Institute[37], European Social Survey[26]*	X				X	R2	R2			R2					X					.81
**Situational risk and resilience**																					
Received empathy[n]			X	X	X				X	X				X	X	R2	X	X	R2	X	.87
Family function[t]	FAD[38]*						X	X	X	X											.91
Social support[u]	European Social Survey[4,26]	X	X			X	X	X			X	X	X			X			X		
Social support	MSPSS[39]*			X	X				X	X				X	X		X	X		X	.92-.97
Community relationships[v]	European Social Survey[4]*	X	X			X	X	X			X	X	X			X			X		
Community resilience[m]	CCRAM10[40]	X				X	R1	R1			R1					X					.92
National resilience[m]	NR-13[41]*	X				X	R1	R1			R1					X					.84
Exposure to terror & war[w]	Kirschenbaum [42], Schiller et al.[43]*	X	X	X	X	X	X	X	X	X	X	X	X	X	X	X	X	X	X	X	
Military service[x]		X	X			X	X	X			X	X	X			X			X		
Bomb shelter access		X				X					X					X			X		
News consumption[y]	European Social Survey[26], Newman et al.[44], Tenenboim–Weinblatt et al. [45]*	X	X	X	X	X	X	X	X	X	X	X	X	X	X	X	X	X	X	X	
Financial impact[z]	Israel Central Bureau of Statistics[27]	X	X	X	X	X	X	X	X	X	X	X	X	X	X	X	X	X	X	X	
Displacement[aa]			C	C	C			C	C	C		C	C	C	C	C	C	C	C	C	
Perception of current situations	Oreg et al.[46]										X	X	X	X	X						.70-90
Disability of self or close others[f,bb]	Myers-JDC-Brookdale Institute [47–49]*															C					
**Public discourse**																					
Criticism versus unity[k]		C	X			C	R2	R2	X	X	R2										.83
Role of government vs citizens[k]	Svallfors [50]*	C				C															.93
National priorities of war[cc]							X	X													
Israel-Hamas negotiations[dd]	Knafo-Noam et al.[51], Petrocelli et al.[52]				X					X					X	X		X			.94
Opinion concerning Hezbollah[ee]																X					
Opinion concerning Iran[ff]				X					X					X		X	X			X	
**General**																					
Open-ended questions[gg]		X	X	X	X	X	X	X	X	X	X	X	X	X	X	X	X	X	X	X	
Demographics[hh]		X		X	X	X			X	X	X			X	X	X	X	X	X	X	
Attention checks[ii]		X	X	X	X	X	X	X	X	X	X	X	X	X	X	X	X	X	X	X	

Note. X indicates that the measure (partial or complete) was included in the relevant sample wave survey. C indicates that questions in the relevant section were conditionally displayed based on participant responses to a filter question. R indicates that questions in the relevant section were randomized across participants. For example, in all samples at the six-month follow-up (except for in Panel A), measures of life satisfaction and primal world beliefs (R1) were randomized against measures of post-traumatic growth and attachment (R2) so that approximately half of the participants in each sample received either pairing of measures, but not both. As a reminder, for ease of reference in the notes below, the six-month follow-up includes T3 of Panel A, T4 of Panels B and C, and T2 of the Community and Student Samples; and the one-year follow-up includes T4 of Panel A, T5 of Panels B and C, and T3 of the Community Sample. Cronbach alphas of each multi-item measure were computed at the first time point it was administered in the Panel Sample (with one exception – alphas for physical and emotional pain were computed on Community Sample data, because this measure

was not administered to Panel Sample participants). When there are multiple subscales, the range of alphas is presented. In select case (e.g., negative affect), the scale was improved in subsequent waves. * indicates that the measure was modified from the original version (e.g., questions may have been adapted, added, or removed). Measures without a cited source are original.

[a] Superscript numbers refer to the reference list.

[b] The item concerning suicidal ideation was not included.

[c] These items were removed from the Community Sample on October 25, 2023.

[d] The items *angry* and *scared* were not included in Panels A and B at T1. They were added to the Community Sample on October 23, 2023. The *bored* item was not included in the six-month or one-year follow-ups.

[e] The scale was reversed (presented from disagreement to agreement, rather than vice versa) in Panels A and C at T2 and in Panels B and C at T3.

[f] This measure was added to the Community Sample on January 18, 2024.

[g] Volunteer work prior to the war was not asked about in Panel A at T2, Panel C at T3, and the six-month or one-year follow-ups. Non-organized volunteer work was not asked about in Panels A and B at T1; it was asked about in the Community Sample beginning on October 17, 2023.

[h] Values were not assessed in the Community Sample from October 25, 2023, through November 22, 2023, due to a technical error. Five additional items were added to the Community Sample-T1 beginning on March 17, 2024.

[i] Items concerning religious community or prayer were not included in the Panel Samples at T1 or in Panel B at T2, and these items were added to the Community Sample on January 18, 2024. At the six-month and one-year follow-ups only, participants were asked about god's protection at the personal level and collective level (either of Israel or Jews, randomly assigned).

[j] Religious change was asked about in the Community Sample beginning on January 18, 2024. Spiritual change was asked about in the Panel Samples only at the six-month and one-year follow-ups, and in the Community Sample beginning on January 21, 2024.

[k] When conditional in the Panel Sample, participants who indicated being in a relationship and having a child between the ages of 9-15 received the emotion regulation assessment, otherwise they were presented (when included) the measures assessing primal world beliefs, criticism versus unity, and role of the government versus civil society.

[l] In the Panel Sample, participants who indicated being in a relationship and having a child between the ages of 9-15 received the emotion regulation assessment (as indicated in note [j]). In Panels A and B at T1, these participants completed the full measure. In Panels A and B at T2 and Panel B at T3, these participants responded to questions concerning relationship quality with partner and child, but not about regulation strategies or motivations pertaining to the self or either relationship. In the Community Sample, we included the regulation motivation items pertaining to the self and (provided the participant indicated being in a relationship) one's partner, from October 16, 2023, through February 4, 2024. In addition, from October 16, 2023, through November 21, 2023, participants in the Community Sample were invited to participate in an additional survey concerning emotional coping, where they were also supplied with resources to assist in coping with negative emotions during the war. This additional survey also included assessments of: (a) self-regulation strategies, (b) regulation strategies and relationship quality pertaining to partners (for those in relationships), and (c) regulation motivation, regulation strategies, and relationship quality pertaining to children (for those with children above age 2). Participants who agreed to participate clicked a link which led them to the additional survey. They were asked to provide the last 5 digits of their ID number in both surveys for the purpose of merging data. The vast majority complied. The data of those who did not provide this information were merged on the basis of the available demographic details in both files (relationship status and child details) and the time stamps of each survey, as follows. First, responses were merged when all demographic details matched, the time-gap between surveys was small, and there were no other possible matches. Next, responses were merged when all demographic details matched, the time-gap between surveys was larger, and there were no other possible matches. When there was more than one possible match, the emotion regulation response was discarded.

[m] These items were removed from the Community Sample on October 25, 2023.

[n] This measure was added to the Community Sample on February 4, 2024.

[o] This item was added across all samples beginning on December 5, 2023.

[p] Panels A and B contain a partial measure (i.e., four of 16 items).

[q] This item was added to the Community Sample on December 4, 2023, and to Panels A and C on December 5, 2023.

[r] In the Community Sample, this item was presented prior to October 25, 2023, from November 22, 2023, through December 5, 2023, and from December 13, 2023, through February 4, 2024.

[s] Overall, 13 items were included across all samples. In Panel B at T2 and T3 and in Panel C at T1 participants were asked separately about Israeli media versus western media (as opposed to media in general), but were not asked about politicians, political parties, the legal system, and academia. In the Community Sample, the items were presented as follows: Some items were removed for good on October 25, 2023 (academia, political parties, politicians, the legal system, and media); other items were also removed on this date, but were returned to the survey on November 22, 2023 (parliament, government, police, military, United Nations, Israeli Security Agency); of these items, two were removed for good on December 5, 2023 (United Nations, Israeli Security Agency), and four were removed for good on February 4, 2024 (parliament, government, police, military). Additionally, in the Community Sample, participants were asked separately about Israeli media versus western media from November 22, 2023, through December 5, 2023.

[t] At T4 and T5, participants were asked about family function during the war, rather than prior to the war (as in T2 and T3).

[u] The item about helpfulness of received support was only presented in Panel A at T2, Panel C at T3, the Community Sample (beginning January 18, 2024), and the Student Sample.

[v] The item assessing whether participants feel they are treated with respect was only asked in Panels A and B at T1 and in the Community Sample through November 22, 2023.

[w] A general item about knowing someone personally who was injured or killed during the security incidents was included in all waves except for the six-month and one-year follow-ups (the term for security incidents in colloquial Hebrew is understood to refer to recent events of terrorism and war). An item about the possibility of getting hurt (due to proximity) in a terror attack or rocket attack was added to the Community Sample on January 18, 2024. Regarding the Panel Sample specifically: an item about participants' city of residence was only included in Panels A and B at T1; items about location during the war outbreak and if it was an affected area were not included in Panel A at T2 or Panel C at T3; an improved item about location during the war outbreak was included at the six-month and one-year follow-ups; items about exposure to rocket attacks and terror attacks prior to the war were included at T1 only; an item about the possibility of getting hurt (due to proximity) in a

terror attack or rocket attack was only included in Panel C at T1; items about terror attacks during the current war were included in all waves; and items about rockets hitting houses were excluded from Panel B at T2 and T3 and from Panel C at T2.

[x] After three days of Community Sample data collection (October 18, 2023) and after Panels A- and B-T1, the following changes were made: An item about personal military service was included (adding to the items about military service of close others), and participants were asked to indicate the region(s) of service of the self and close others across the board (in Panels A and B at T1 participants indicated the region of service for each close other separately, and participants in the Community Sample were not asked about region of service prior to this point). Beginning on January 18, 2024, participants were only presented the entire measure if they indicated that they or close others are currently serving in the military.

[y] Beginning on October 20, 2023, an improved item assessing news consumption prior to the war was administered. Beginning on January 18, 2024, news consumption during the war was assessed separately for early during the war (though not assessed at the six-month and one-year follow-ups) and during recent weeks. An expanded measure (e.g., assessing consumption of specific outlets and via various platforms) was presented only in Panel C at T2.

[z] Concern that one will be financially affected was assessed in all waves, however, whether one was already affected was included beginning on October 22, 2023. Additional specifics of financial impact were assessed beginning on November 12, 2023, in the Panel Samples, and on November 22, 2023, in the Community Sample. Participants' ability to cover their expenses was assessed beginning on December 5, 2023.

[aa] Items about displacement details were only presented to those who indicated that they were displaced. This measure was included in the Community Sample beginning on November 22, 2023. On March 17, 2024, in the Community Sample, and at the six-month and one-year follow-ups in all groups, options were added to the question concerning the length of displacement to allow for longer durations.

[bb] Items about the details of personal or dependents' disabilities were only presented to those who indicated that they have a disability or care for someone with a disability, respectively.

[cc] The items were presented in a random order. Due to a clerical error, the instructions were also randomized (i.e., not all participants received the instructions first).

[dd] In the Community Sample at T1, items about the first Israel-Hamas negotiations were included from November 21, 2023, through December 4, 2023, and items about the second Israel-Hamas negotiations were included from February 1, 2024. In addition, items about opinion confidence and clarity were included beginning on March 17, 2024. These questions (opinion, and confidence and clarity) concerned an updated version of the hostage negotiations at the one-year follow-up (in both the Community and Panel Samples).

[ee] This item was presented to participants beginning on February 28, 2024.

[ff] These items were added to the Community Sample-T1 on April 16, 2024.

[gg] A total of eight open-ended questions were included at various junctures during the war. Questions concerning personal change due to the war, how individuals cope, and requests of the local authorities were only presented in some of the Panel Sample waves. Student Sample participants were not asked about requests of the local authorities or what they would tell country leadership if they had the chance. Community Sample participants were asked about personal change due to the war beginning October 25, 2023, about requests of the local authorities from October 17, 2023, through December 5, 2023, and about how they cope beginning January 18, 2024.

[hh] Employment details and children's ages were not assessed in Panels A and B at T1. These items were added to the Community Sample beginning on October 17, 2023, and October 21, 2023, respectively. In the Student Sample, participants were allowed to indicate more than one employment status option. At the six-month and one-year follow-ups, items assessing relatively stable constructs were excluded (e.g., birth country).

[ii] Due to a technical error, the second attention check was not displayed to participants in Panel B at T2 and T3.

Additional Method Details

Community and Student Sample recruitment

In the Community and Students Samples, recruitment for subsequent waves relied on participants' provision of valid email addresses or WhatsApp numbers during the first wave of data collection. Valid email address *n*s presented in the main text were computed based on data provided by our recruitment platform (QuestionPro for the relevant waves) concerning the number of recruitment emails that were successfully delivered (i.e., not invalid, bounced). Valid WhatsApp *n*s were computed as the amount of phone numbers provided minus those who could not be found on WhatsApp or people who said they did not previously participate in our study (i.e., wrong numbers).

Four participants who participated in the Community Sample at T1 did not have a time stamp (seemingly due to technical error), and we therefore do not know when they participated. Recruitment to T2 was contingent on having participated prior to April 6, 2024, in addition to providing valid contact information. Of the four participants who did not have a timestamp, only one provided valid contact information. This participant was recruited to T2 even though we do not know if T1 participation fit the data-related inclusion criteria (i.e., participation prior to April 6, 2024). Nevertheless, this participant did not respond at T2 (or T3). The eligibility and response rates reflect this strategy.

Participation inclusion and data retention

Two additional responses were removed from the Community Sample: one from T2 and one from T3. These responses were from individuals who did not participate at T1 but accessed the survey via a shared link of another participant.

References

1. Central Bureau of Statistics. *Ages 20 and above, by level of religiosity and selected characteristics (Hebrew)*. https://www.cbs.gov.il/he/publications/LochutTlushim/%D7%9C%D7%95%D7%97%D7%95%D7%AA%20%D7%A9%D7%A0%D7%AA%D7%95%D7%9F/st28_06x.pdf (2023).
2. Kroenke, K., Spitzer, R. L., Williams, J. B. & Löwe, B. An Ultra-Brief Screening Scale for Anxiety and Depression: The PHQ-4. *Psychosomatics* 50, 613–621 (2009).
3. Löwe, B., Kroenke, K., Herzog, W. & Gräfe, K. Measuring depression outcome with a brief self-report instrument: Sensitivity to change of the Patient Health Questionnaire (PHQ-9). *J Affect Disord* 81, 61–66 (2004).
4. European Social Survey European Research Infrastructure (ESS ERIC). *ESS6 - Integrated File, Edition 2.6 [Data Set]. Sikt - Norwegian Agency for Shared Services in Education and Research* (2023) doi:10.21338/ess6e02_6.
5. Löwe, B. *et al.* Validation and Standardization of the Generalized Anxiety Disorder Screener (GAD-7) in the General Population. *Med Care* 266–274 (2008).
6. Norman, S. B., Hami Cissell, S., Means-Christensen, A. J. & Stein, M. B. Development and validation of an Overall Anxiety Severity and Impairment Scale (OASIS). *Depress Anxiety* 23, 245–249 (2006).
7. Rees, G., Savahl, S., Lee, B. J. & Casas, F. *Children's Views on Their Lives and Well-Being in 35 Countries: A Report on the Children's Worlds Project, 2016-19 (ISCWeB)*. (2020).
8. Feldman Barrett, L. & Russell, J. A. Independence and Bipolarity in the Structure of Current Affect. *J Pers Soc Psychol* 74, 967 (1998).
9. Gross-Manos, D., Shimoni, E. & Ben-Arieh, A. Subjective Well-Being Measures Tested with 12-Year-Olds in Israel. *Child Indic Res* 8, 71–92 (2015).
10. Blevins, C. A., Weathers, F. W., Davis, M. T., Witte, T. K. & Domino, J. L. The Posttraumatic Stress Disorder Checklist for DSM-5 (PCL-5): Development and Initial Psychometric Evaluation. *J Trauma Stress* 28, 489–498 (2015).
11. Weathers, F. W. *et al.* The PTSD checklist for DSM-5 (PCL-5). (2013).
12. Cook, K. F. *et al.* Pain assessment using the NIH Toolbox. *Neurology* 80, S49–S53 (2013).
13. Gilam, G. *et al.* Negative affect-related factors have the strongest association with prescription opioid misuse in a cross-sectional cohort of patients with chronic pain. *Pain Medicine (United States)* 21, E127–E138 (2020).
14. Schwartz, S. H. A Repository of Schwartz Value Scales with Instructions and an Introduction. *Online Readings in Psychology and Culture* 2, (2021).
15. Fetzer Institute. *Multidimensional Measurement of Religiousness/Spirituality for Use in Health Research: A Report of the Fetzer Institute/National Institute on Aging Working Group.* (1999).
16. Pargament, K. I., Smith, B. W., Koenig, H. G. & Perez, L. Patterns of Positive and Negative Religious Coping with Major Life Stressors. *J Sci Study Relig* 37, 710–724 (1998).
17. Abu-Raiya, H., Sasson, T., Pargament, K. I. & Rosmarin, D. H. Religious Coping and Health and Well-Being among Jews and Muslims in Israel. *International Journal for the Psychology of Religion* 30, 202–215 (2020).

18. Clifton, J. D. W. & Yaden, D. B. Brief Measures of the Four Highest-Order Primal World Beliefs. *Psychol Assess* 33, 1267–1273 (2021).
19. Tamir, M. *et al.* Emotion Regulation Strategies and Psychological Health Across Cultures. *American Psychologist* 79, 748–764 (2024).
20. Shahar, G. The heroic self: Conceptualization, measurement, and role in distress. *Int J Cogn Ther* 6, 248–264 (2013).
21. Blatt, S. J., Schaffer, C. E., Bers, S. A. & Quinlan, D. M. Psychometric properties of the Adolescent Depressive Experiences Questionnaire. *J Pers Assess* 59, 82–98 (1992).
22. Schwarzer, R., & Jerusalem, M. (1995). Generalized Self-Efficacy scale. In J. Weinman, S. Wright, & M. Johnston, Measures in health psychology: A user's portfolio. Causal and control beliefs (pp. 35-37). Windsor, UK: NFER-NELSON.
23. Soto, C. J. & John, O. P. The next Big Five Inventory (BFI-2): Developing and assessing a hierarchical model with 15 facets to enhance bandwidth, fidelity, and predictive power. *J Pers Soc Psychol* 113, 117–143 (2017).
24. Lafontaine, M. F. *et al.* Selecting the best items for a short-form of the experiences in close relationships questionnaire. *European Journal of Psychological Assessment* 32, 140–154 (2016).
25. Aron, A., Aron, E. N. & Smollan, D. Inclusion of Other in the Self Scale and the Structure of Interpersonal Closeness. *J Pers Soc Psychol* 63, 596–612 (1992).
26. European Social Survey European Research Infrastructure (ESS ERIC). *ESS Round 10 - 2020. Democracy, Digital Social Contacts*. *Sikt - Norwegian Agency for Shared Services in Education and Research* (2023) doi:10.21338/NSD-ESS10-2020.
27. Central Bureau of Statistics. The Social Survey (in Hebrew). https://www.cbs.gov.il/he/Surveys/Pages/%D7%A1%D7%A7%D7%A8-%D7%97%D7%91%D7%A8%D7%AA%D7%99.aspx (2024).
28. Davis, M. H. A multidimensional approach to individual differences in empathy. *JSAS Catalog of Selected Documents in Psychology* (1980).
29. Chiu, C. Y., Hong, Y. Y. & Dweck, C. S. Lay Dispositionism and Implicit Theories of Personality. *J Pers Soc Psychol* 73, 19 (1997).
30. Levontin, L., Nakash, O. & Danziger, S. It takes two to self-disclose: Incremental theorists facilitate others' self-disclosure more than do entity theorists. *J Pers* 87, 1264–1276 (2019).
31. Cann, A. *et al.* A short form of the posttraumatic growth inventory. *Anxiety Stress Coping* 23, 127–137 (2010).
32. Tedeschi, R. G. & Calhoun, L. G. The Posttraumatic Growth Inventory: Measuring the Positive Legacy of Trauma. *J Trauma Stress* 9, 455–471 (1996).
33. Costin, V. & Vignoles, V. L. Meaning is about mattering: Evaluating coherence, purpose, and existential mattering as precursors of meaning in life judgments. *J Pers Soc Psychol* 118, 864 (2020).
34. Scheier, M. F., Carver, C. S. & Bridges, M. W. Distinguishing Optimism From Neuroticism (and Trait Anxiety, Self-Mastery, and Self-Esteem): A Reevaluation of the Life Orientation Test. *J Pers Soc Psychol* 67, 1063–1078 (1994).
35. Roccas, S., Sagiv, L., Schwartz, S., Halevy, N. & Eidelson, R. Toward a unifying model of identification with groups: Integrating theoretical perspectives. *Personality and Social Psychology Review* 12, 280–306 (2008).

36. Douglass, S. & Umaña-Taylor, A. J. A Brief Form of the Ethnic Identity Scale: Development and Empirical Validation. *Identity* 15, 48–65 (2015).
37. Hermann, T., Anabi, O., Kaplan, Y. & Sapozhnikova, I. O. *The Israeli Democracy Index 2022 (Hebrew). The Israel Democracy Institute*. (2023).
38. Epstein, N. B., Baldwin, L. M. & Bishop, D. S. The McMaster Family Assessment Device. *J Marital Fam Ther* 9, 171–180 (1983).
39. Zimet, G. D., Dahlem, N. W., Zimet, S. G. & Farley, G. K. The multidimensional scale of perceived social support. *J Pers Assess* 52, 30–41 (1988).
40. Leykin, D., Lahad, M., Cohen, O., Goldberg, A. & Aharonson-Daniel, L. Conjoint Community Resiliency Assessment Measure-28/10 Items (CCRAM28 and CCRAM10): A Self-report Tool for Assessing Community Resilience. *Am J Community Psychol* 52, 313–323 (2013).
41. Kimhi, S. & Eshel, Y. Measuring national resilience: A new short version of the scale (NR-13). *J Community Psychol* 47, 517–528 (2019).
42. Kirschenbaum, A. Terror, adaptation and preparedness: A trilogy for survival. *J Homel Secur Emerg Manag* 3, (2006).
43. Schiller, M., Pinus, M., Hammen, C. C. & Shahar, G. Effects of Psychological Distress and Exposure to Terror-Related Stress on the Self in Emerging Adulthood. *Int J Cogn Ther* 12, 242–259 (2019).
44. Newman, N. R., Fletcher, R., Robertson, C., Ross Arguedas, A. & Nielsen, R. K. Reuters Institute digital news report 2024. *Reuters Institute for the Study of Journalism.* https://reutersinstitute.politics.ox.ac.uk/digital-news-report/2024 (2019).
45. Tenenboim-Weinblatt, K., Baden, C., Aharoni, T. & Overbeck, M. Affective forecasting in elections: A socio-communicative perspective. *Hum Commun Res* 48, 553–566 (2022).
46. Oreg, S., Edwards, J. A. & Rauthmann, J. F. The Situation Six: Uncovering Six Basic Dimensions of Psychological Situations From the Hebrew Language. *J Pers Soc Psychol* 118, 835–863 (2020).
47. Barlev, L., Namer Furstenberg, R. & Gadge, N. *Children with Disabilities in Israel: A National Study. RR-885-21. Myers-JDC-Brookdale Institute. (Hebrew)*. (2021).
48. Nagar Eidelman, R., Barlev, L., Namer Furstenberg, R. & Golovenzitz, E. *Participation in Society of People with Disabilities. RR-012-24. Myers-JDC-Brookdale Institute. (Hebrew)*. (2024).
49. Hercowitz-Amir, A., Ayali, T., Nagar Eidelman, R., Barlev, L. & Resnizky, S. *Family Caregivers of Older Adults and People with Disabilities During the Initial Months of the Israel-Hamas War. RR-023-24. Myers-JDC-Brookdale Institute. (Hebrew)*. (2024).
50. Svallfors, S. Policy feedback, generational replacement, and attitudes to state intervention: Eastern and Western Germany, 1990–2006. *European Political Science Review* 2, 119–135 (2010).
51. Knafo-Noam, A. *et al.* The role of personal values in opinion formation regarding a high-stakes continually evolving topic: The Hamas-Israel hostage deal negotiations. *American Psychologist.* (2025).
52. Petrocelli, J. V., Tormala, Z. L. & Rucker, D. D. Unpacking attitude certainty: Attitude clarity and attitude correctness. *J Pers Soc Psychol* 92, 30–41 (2007).
